# Subthreshold activation of the melanocortin system causes generalized sensitization to anorectic agents in mice

**DOI:** 10.1172/JCI178250

**Published:** 2024-07-15

**Authors:** Naima S. Dahir, Yijun Gui, Yanan Wu, Patrick R. Sweeney, Alix A.J. Rouault, Savannah Y. Williams, Luis E. Gimenez, Tomi K. Sawyer, Stephen T. Joy, Anna K. Mapp, Roger D. Cone

**Affiliations:** 1 Life Sciences Institute,; 2 Department of Molecular and Integrative Physiology, and; 3 Department of Molecular, Cellular and Developmental Biology, University of Michigan, Ann Arbor, Michigan, USA.; 4 Department of Molecular and Integrative Physiology, University of Illinois, Urbana-Champaign, Illinois, USA.; 5 Courage Therapeutics, Newton, Massachusetts, USA.; 6 Department of Chemistry, School of Literature, Science, and the Arts, University of Michigan, Ann Arbor, Michigan, USA.

**Keywords:** Metabolism, Obesity

## Abstract

The melanocortin-3 receptor (MC3R) regulates GABA release from agouti-related protein (AgRP) nerve terminals and thus tonically suppresses multiple circuits involved in feeding behavior and energy homeostasis. Here, we examined the role of the MC3R and the melanocortin system in regulating the response to various anorexigenic agents. The genetic deletion or pharmacological inhibition of the MC3R, or subthreshold doses of an MC4R agonist, improved the dose responsiveness to glucagon-like peptide 1 (GLP1) agonists, as assayed by inhibition of food intake and weight loss. An enhanced anorectic response to the acute satiety factors peptide YY (PYY3-36) and cholecystokinin (CCK) and the long-term adipostatic factor leptin demonstrated that increased sensitivity to anorectic agents was a generalized result of MC3R antagonism. We observed enhanced neuronal activation in multiple hypothalamic nuclei using Fos IHC following low-dose liraglutide in MC3R-KO mice (*Mc3r^–/–^*), supporting the hypothesis that the MC3R is a negative regulator of circuits that control multiple aspects of feeding behavior. The enhanced anorectic response in *Mc3r^–/–^* mice after administration of GLP1 analogs was also independent of the incretin effects and malaise induced by GLP1 receptor (GLP1R) analogs, suggesting that MC3R antagonists or MC4R agonists may have value in enhancing the dose-response range of obesity therapeutics.

## Abstract 2

Genetic or pharmacological blockade of the melanocortin-3 receptor, or subthreshold stimulation of the melanocortin-4 receptor, chemosensitizes animals to multiple anorectic drugs and hormones.

## Introduction

The central melanocortin system is fundamental in regulating feeding behavior and energy homeostasis. Mutations in the melanocortin-4 receptor (MC4R) or genetic deletion of *Mc4r* results in hyperphagia, hyperinsulinemia, and obesity (1–3). In contrast, *Mc3r* deletion does not produce hyperphagia or hyperinsulinemia in ad libitum–fed animals (4, 5) However, the MC3R acts as a negative regulator of the MC4R system, controlling responses of the melanocortin system to both internal and external challenges (6–10), controlling the upper and lower boundaries of energy homeostasis (9), and providing sexually dimorphic inputs to the system (8, 10, 11). For example, MC3R is essential in promoting normal compensatory refeeding and neuroendocrine responses to fasting (7, 8, 10, 12). The MC3R accomplishes this by regulating GABA release onto MC4R neurons from presynaptic MC3R expressed on agouti-related protein (AgRP) neurons (9, 12) and controlling the activation of AgRP neurons (13). Furthermore, the MC3R is requisite for normal growth (4, 5) and reproductive function, with mutations leading to reduced linear growth, lean mass, and late onset of puberty in mice and humans (2).

We have previously examined the effect of the MC3R on MC4R-expressing paraventricular hypothalamic (PVH) neurons and demonstrated that MC3R blockade enhances the magnitude and duration of the inhibition of food intake in response to a single dose of the MC4R agonist LY2112688 (9). The MC3R is expressed in nearly all AgRP neurons, and the AgRP neurons are known to broadly affect multiple aspects of feeding behavior and suppress competing behavioral states, including fear, alarm, and thirst. For example, we have demonstrated that deletion of the MC3R increases sensitivity to anorexia induced by restraint and social isolation (10). Thus, MC3R activity may be found to broadly regulate responsiveness to anorectic stimuli. Here, we examined the ability of the MC3R, and the downstream effects of the MC4R, to regulate responsiveness to a variety of anorexigenic agents, from acute satiety factors like CCK and PYY_3–36_ to the long-term adipostatic factor leptin, as well as to leading glucagon-like peptide-1–based (GLP1-based) therapeutic agents like liraglutide and tirzepatide.

## Results

### Disruption of MC3R signaling increases sensitivity to GLP1R agonists.

**Figure 1:**
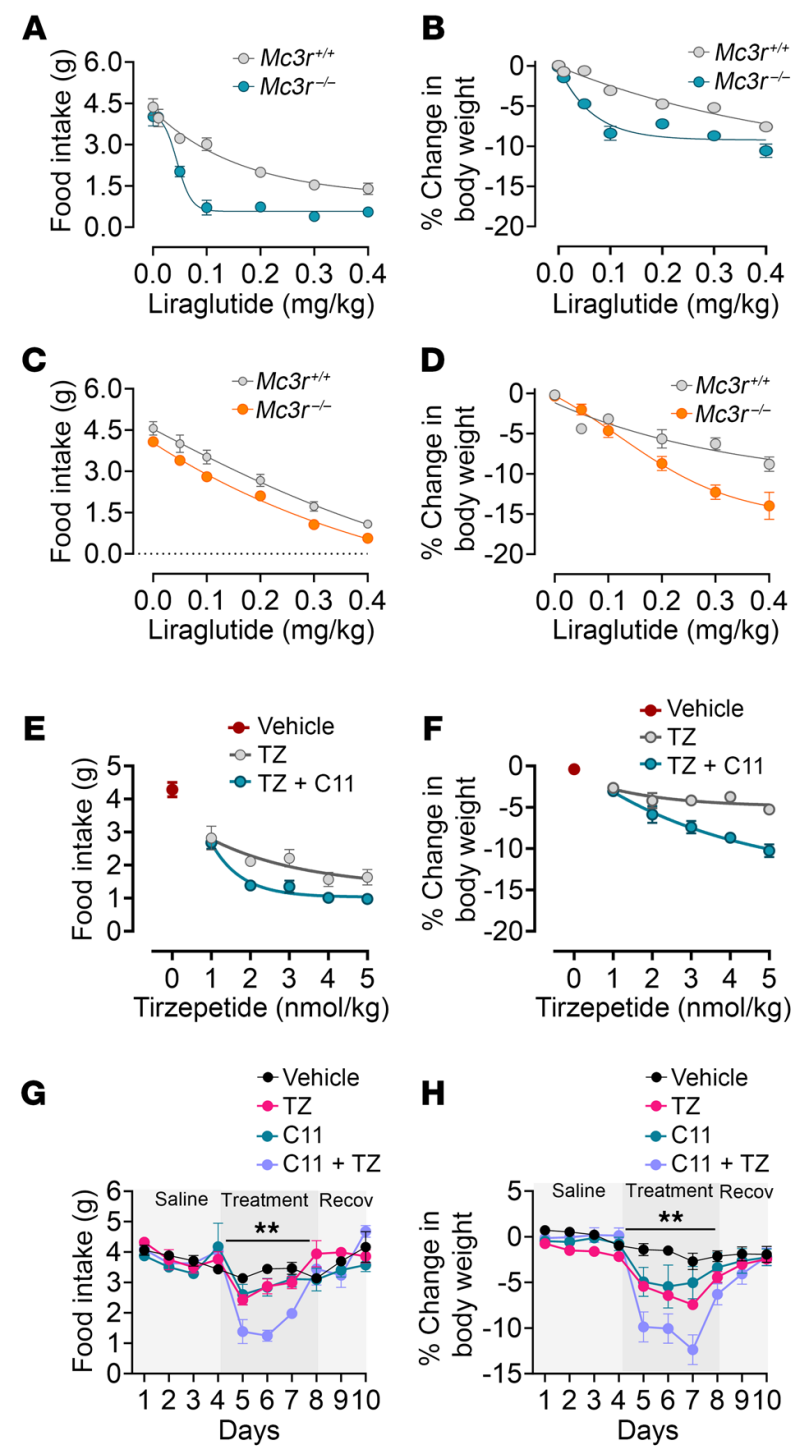
MC3R loss increases responsiveness to GLP1 drugs. Liraglutide (0.05–0.4 mg/kg) administration resulted in more significant inhibition of (**A**) food intake and (**B**) weight loss in *Mc3r^–/–^* male mice compared with *Mc3r^+/+^* mice in a dose-dependent manner after 24 hours (*n* = 7–8/group). (**C**) Liraglutide-induced feeding and (**D**) body weight changes of *Mc3r^+/+^* and *Mc3r^–/–^* female mice (*n* = 7–8/group; 0.05–0.4 mg/kg). Tirzepatide (TZ) (1–4 nmol/kg) and coadministration of tirzepatide and C11-induced (**E**) feeding responses and (**F**) body weight changes of WT male mice (vehicle, *n* = 10; all other groups, *n* = 8) at 24 hours after injection. (**G**) Twenty-four-hour feeding and (**H**) body weight changes after chronic injections of tirzepatide (2 nmol/kg), C11 (0.5 nmol), tirzepatide plus C11, and vehicle. Data are expressed as the mean ± SEM, and statistical tests were performed by 2-way ANOVA with Tukey’s test for post hoc analysis (**G**–**H**). For all the dose-response curve data, a repeated-measures 2-way ANOVA was corrected for multiple comparisons using the Tukey-Kramer method for each time point, and data were fitted with 4 parameters: nonlinear fit, **P < 0.01.

Previously, we reported that deletion of the MC3R (*Mc3r^–/–^*) in male and female mice results in enhanced acute inhibition of food intake in response to a single dose of the glucagon-like peptide-1 receptor (GLP1R) agonist liraglutide (10). We tested the effect of various concentrations of liraglutide in mice lacking *Mc3r* and their WT counterparts. In this testing paradigm, we administered concentrations of 0.05–0.4 mg/kg liraglutide at least 30 minutes before the onset of the dark cycle. We manually measured a single time point (24-hour) for food intake (spillage included) and body weight change in male and female mice. As expected, liraglutide reduced food intake and body weight in WT male (Figure 1, A and B) and female (Figure 1, C and D) mice in a concentration-dependent manner. In the *Mc3r^–/–^* mice, we found an increased dose sensitivity to liraglutide-induced suppression of food intake and weight loss 24 hours after administration. Unlike WT mice, *Mc3r^–/–^* mice exhibited reduced food intake and weight loss in response to low concentrations of liraglutide (0.05 mg/kg), indicating hypersensitivity to the compound. Next, we assessed the sensitivity of *Mc3r^–/–^* mice to other GLP1R agonists. We tested the effect of semaglutide, a secondary GLP1 drug shown to have a longer halflife than liraglutide, thereby increasing both the incretin and anorectic duration of drug action (14). We tested male mice and found that, like liraglutide, deletion of MC3R led to increased responsiveness to semaglutide; *Mc3r^–/–^* mice responded more to both the anorectic effects and weight loss following semaglutide treatment compared with the WT mice, illustrating that this hypersensitivity was in response to multiple GLP1R agonists (Supplemental Figure 1, A and B; supplemental material available online with this article; https://doi.org/10.1172/JCI178250DS1).

We have previously shown that the MC3R peptide antagonist compound 11 (C11) (15) can reliably inhibit the activity of the MC3R and that coadministration of C11 and liraglutide can further lower the body weight of WT mice (10). We repeated the same experiment and additionally used *Mc3r^–/–^* mice to illustrate the specificity of C11. We found that pharmacological inhibition of the MC3R (C11; 1 nmol/1 μL, i.c.v.) and liraglutide, together, resulted in profound weight loss and decreased food intake over 8-hour, 12-hour, and 24-hour periods (Supplemental Figure 2). Furthermore, no effect was seen in mice with MC3R deletion, validating the pharmacological specificity of C11.

Next, we examined the weight loss and anorectic effects of MC3R pharmacological inhibition in response to increasing doses of tirzepatide. This diabetes drug acts as a dual agonist for GLP1R and the glucose-dependent insulinotropic polypeptide (GIP) in humans (16, 17) but acts as a potent GLP1R agonist in rodents (18). Administration of tirzepatide (1–5 nmol/kg, s.c.) to WT mice decreased food intake and promoted weight loss in male mice (Figure 1, E and F), measured 24 hours after administration. Furthermore, coadministration of C11 (1 nmol/1 μL, i.c.v.) and varying dosages of tirzepatide profoundly decreased 24-hour food intake and 24-hour body weight after a single injection of both compounds (Figure 1, E and F). To determine whether chronic administration of tirzepatide and C11 further promotes the hypersensitivity effect, 24-hour food intake and the body weight of male mice administered C11 (0.5 nmol, i.c.v.), tirzepatide ( 2 nmol/kg, s.c.), C11 and tirzepatide, or vehicle (DMSO, i.c.v. and saline, s.c.) were measured. The results for 4 days of vehicle treatment, followed by treatment for 3 days, and the recorded recovery for an additional 3 days are shown in Figure 1, G and H. Administration of C11 and tirzepatide alone showed comparable effects on weight loss and decreased food intake compared with mice in the vehicle-treated groups. However, administration of both compounds led to a greater inhibition of food intake and weight loss than did either compound alone, demonstrating a higher efficacy of tirzepatide with MC3R antagonism in a chronic treatment model.

### MC3R deletion does not increase the incretin effects or malaise induced by liraglutide.

**Figure 2:**
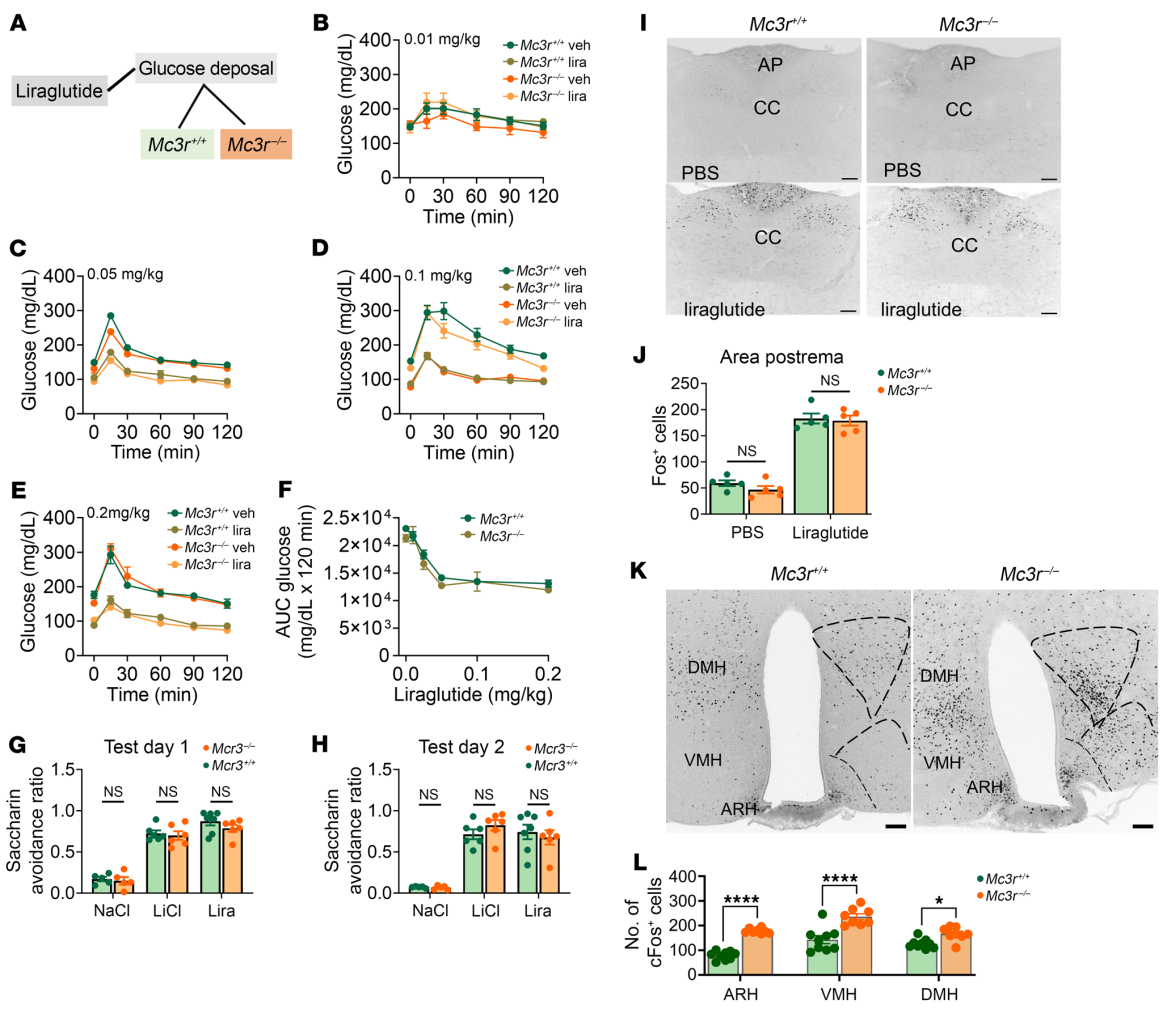
*Mc3r* deletion has no effect on the incretin activity or malaise associated with liraglutide. (**A**–**F**) Glucose levels and AUC before and after oral administration of glucose (1 g/kg) after liraglutide (lira) (0.01–0.2 mg/kg) or vehicle (veh) treatment in *Mc3r^+/+^* and *Mc3r^–/–^* male mice (*n* = 6–7/group). CTA test day 1 (**G**) and test day 2 (**H**) after liraglutide administration in *Mc3r^+/+^* and *Mc3r^–/–^* male mice. (**I**) Representative images from the AP showing Fos IHC after saline or liraglutide injection from *Mc3r^+/+^* and *Mc3r^–/–^* male mice. CC, central canal. Scale bars: 100 μm. (**J**) Quantification of cells expressing Fos after saline or liraglutide injection in *Mc3r^+/+^* and *Mc3r^–/–^* male mice. (**K**) Representative images from the hypothalamus showing Fos IHC after liraglutide treatment in *Mc3r^+/+^* and *Mc3r^–/–^* mice. Scale bars: 100 μm. (**L**) Quantification of cells expressing Fos in the ARH, VMH, and DMH after liraglutide treatment. Statistical analysis was done by 2-tailed Student’s *t* test, *P < 0.05, and ****P < 0.0001 (**J**, **G**, **H**, and **L**).

GLP1 analogs have been historically associated with their incretin effects, stimulating insulin secretion and suppressing glucagon secretion in hyperglycemic or euglycemic states (19). GLP1R analogs approved by the FDA are reliably used to treat type 2 diabetes and regulate glucose homeostasis (20). We tested whether *Mc3r^–/–^* mice also exhibited increased sensitivity to the incretin effects of GLP1 analogs by measuring blood glucose in a glucose tolerance test (GTT) (Figure 2A). WT and *Mc3r^–/–^* mice were fasted and injected with varying doses of liraglutide (0.01–0.2 mg/kg, s.c.) or vehicle (PBS, s.c.). After 6 hours of fasting, the glucose responses were measured following a 1 g/kg oral glucose bolus. However, we found no effect of genotype; both *Mc3r^+/+^* and *Mc3r^–/–^* mice responded similarly to the oral GTT (Figure 2, B–F). Furthermore, we subjected mice to varying glucose boluses (0.5–2 g/kg, oral) in response to liraglutide (0.2 mg/kg, s.c.) and found no effect of genotype; both *Mc3r^+/+^* and *Mc3r^–/–^* mice responded similarly to the oral GTT (Supplemental Figure 3, A–C). Glucose disposal with liraglutide was comparably better than with vehicle in both genotype groups (Supplemental Figure 3, A–C).

The most reported adverse effects of GLP1R agonists are gastrointestinal; patients report increased emetic responses (21–24), as well as exacerbation of gastroparesis (25). To test whether the observed hypersensitive anorectic responses to GLP1 analogs were accompanied by increased malaise, we tested whether liraglutide triggered increased conditioned taste aversion in *Mc3r^–/–^* mice, as previously described (23, 26). Low concentrations of liraglutide that resulted in decreased food intake and promoted weight loss (0.05 mg/kg) in *Mc3r^–/–^* mice were paired with the non-nutritive sweetener saccharin (0.1%). A second group of mice received saccharin paired with LiCl (150 mM) to induce gastric malaise. A third group of mice received saccharin paired with saline (150 mM NaCl). Mice underwent training for 2 days, conditioning for 2 days, and testing for 2 days. *Mc3r^+/+^* and *Mc3r^–/–^* mice showed low avoidance for saccharin when paired with saline and high avoidance when paired with either the liraglutide or LiCl. We found that genetic deletion of *Mc3r* did not increase the conditioned taste aversion associated with administering liraglutide (Figure 2, G and H). GLP1 agonists are also known to activate neurons in the area postrema (AP) at doses that induce conditioned taste aversion (CTA) (27). Deletion of the MC3R was not observed to increase activation of AP neurons in response to 0.1 mg/kg liraglutide (Figure 2, I and J). In contrast, a profound increase in neuronal activation following liraglutide treatment was seen in hypothalamic feeding circuits in the ventromedial hypothalamus (VMH), dorsomedial hypothalamus (DMH), and arcuate nucleus of the hypothalamus (ARH) of the *Mc3r^–/–^* mice relative to WT mice (Figure 2, K and L). Collectively, these data suggest that the observed anorectic hypersensitization of GLP1 analogs in *Mc3r^–/–^* mice was independent of emetic responses.

### MC3R deletion produces increased sensitivity to diverse anorectic hormones.

**Figure 3:**
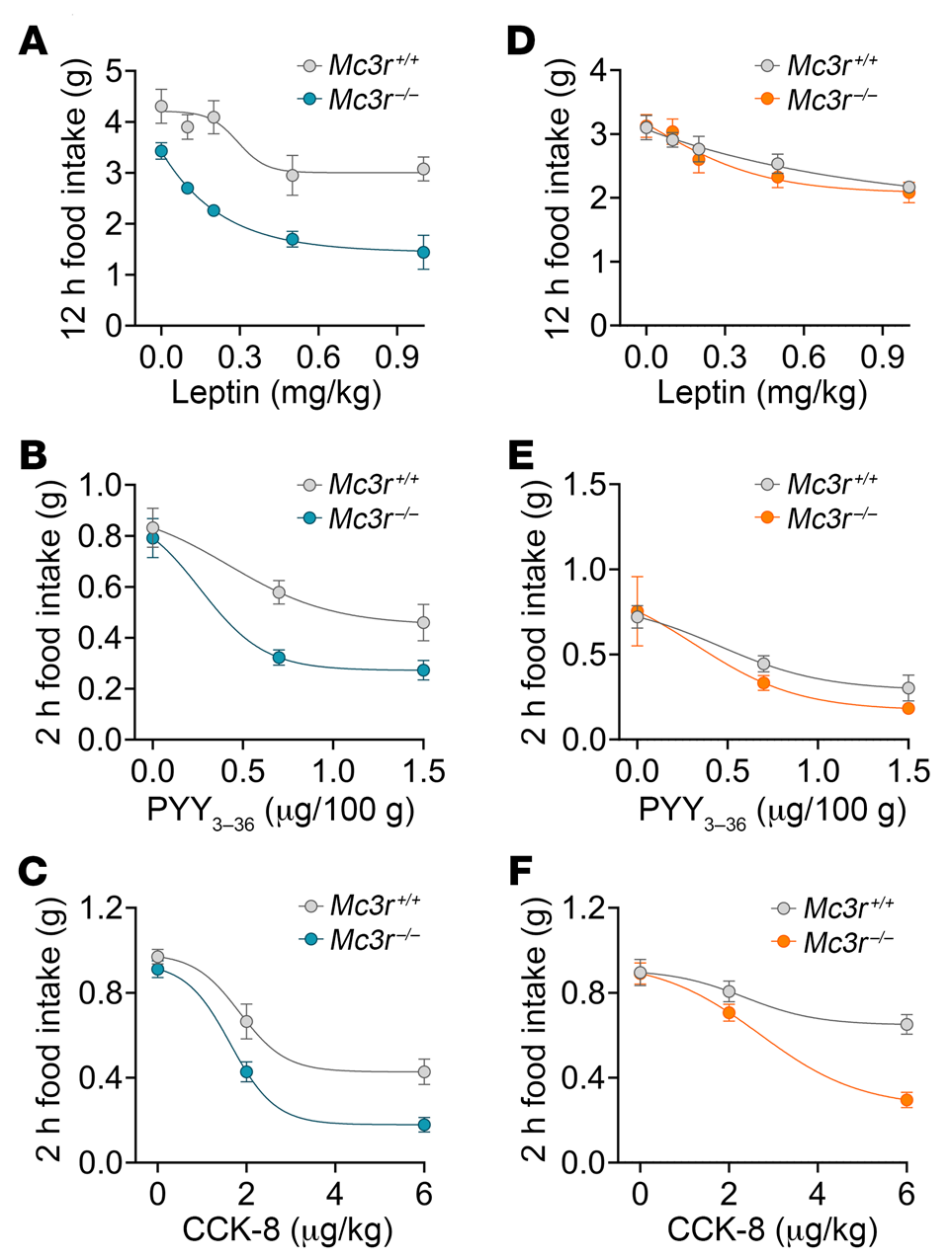
Deletion of *Mc3r* results in generalized enhanced sensitivity to anorectic hormones. Nocturnal feeding in response to leptin (0.1–1 mg/kg) 12 hours after injection (vehicle, *n* = 11, *n* = 6/group) in *Mc3r^+/+^* and *Mc3r^–/–^* (**A**) male and (**D**) female mice. Acute dark-phase feeding after administration of PYY_3–36_ (*n* = 7/group) in *Mc3r^+/+^* and *Mc3r^–/–^* (**B** ) male and (**E**) female mice. Acute dark-phase feeding after administration of CCK (*n* = 7–8/group) in *Mc3r^+/+^* and *Mc3r^–/–^* (**C** ) male and (**F**) female mice. Data represent the mean ± SEM. Statistical analysis was done by 2-way, repeated-measures ANOVA with Tukey’s post hoc test (**A**–**F**).

We next sought to determine whether the role of the MC3R in sensitivity to GLP1 agonists is unique to this family of hormones or more generalizable. We first tested the effect of the long-term adipostatic hormone leptin, a hormone produced by the adipose tissue in proportion to fat stores (28). Given that *Mc3r^–/–^* mice display late-onset weight gain and hyperleptinemia (7, 13), these mice should theoretically exhibit leptin resistance. In contrast, *Mc3r^–/–^* mice also exhibited increased anorectic sensitivity to leptin; low doses of leptin, which otherwise did not affect food intake in WT mice, showed robust anorectic activity in male *Mc3r^–/–^* mice (Figure 3A). The effect was not observed in female mice (Figure 3D), although the estrous cycle, an important determinant of daily food intake and leptin levels (29, 30), was not synchronized in this study. We next tested gut hormones known to act acutely as satiety factors, including a form of the peptide YY (PYY_3–36_) and cholecystokinin (CCK), both secreted by enteroendocrine cells in the small intestine. We found that male and female *Mc3r^–/–^* mice had increased responses to PYY_3–36_ in a dose-dependent manner over a 2-hour nocturnal feeding period (Figure 3, B and E). Similarly, administration of CCK produced an increased anorectic response in male and female *Mc3r^–/–^* mice compared with the WT mice in a dose-dependent manner (Figure 3, C and F).

### Relative contributions of the MC3R and MC4R to liraglutide sensitivity.

**Figure 4:**
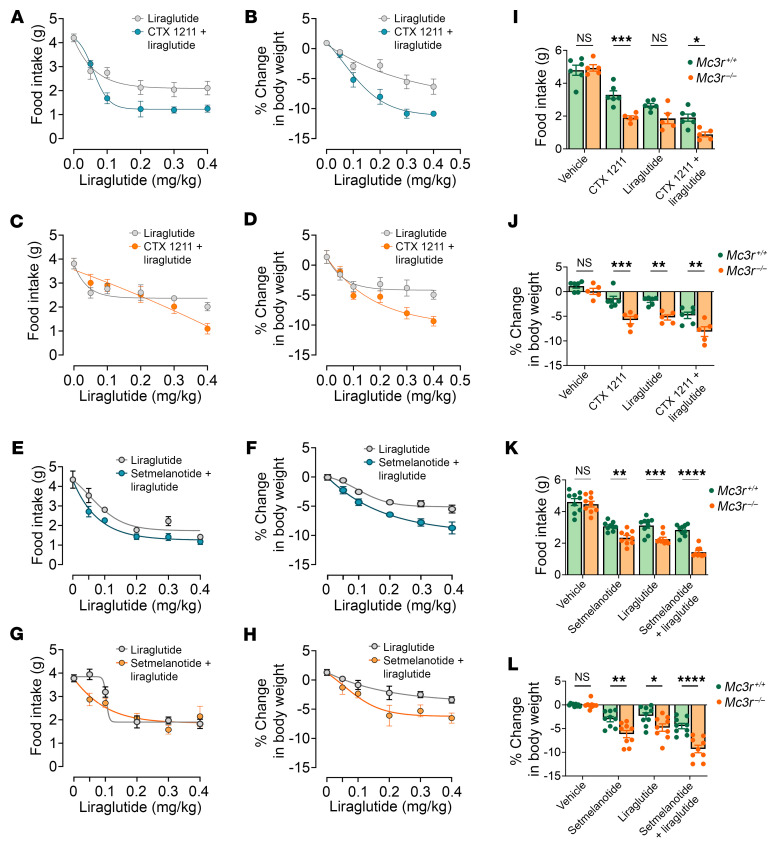
*Mc3r* deletion enhances the ability of an MC4R agonist to increase sensitivity to liraglutide. (**A**, **C**, **E**, and **G**) Twenty-four-hour food intake and (**B**, **D**, **F**, and **H**) body weight changes in response to CTX 1211 or setmelanotide (2 mg/kg, i.p., *n* = 8/group) or to liraglutide alone (0.05–0.4 mg/kg, s.c., *n* = 8), as indicated in male and female mice. (**I** and **K**) Twenty-four-hour food intake and (**J** and **L**) body weight changes in response to CTX 1211 (1.5 mg/kg, i.p., *n* = 5–6) or setmelanotide (1.5 mg/kg, i.p., *n* = 9) alone, liraglutide alone (0.2 mg/kg, s.c.), liraglutide plus CTX 21 or setmelanotide, and vehicle in *Mc3r^+/+^* and *Mc3r^–/–^* mice. Data represent the mean ± SEM. *P < 0.05, **P < 0.01, ***P < 0.001, and ****P < 0.0001, by 2-way, repeated-measures ANOVA.

**Figure 5:**
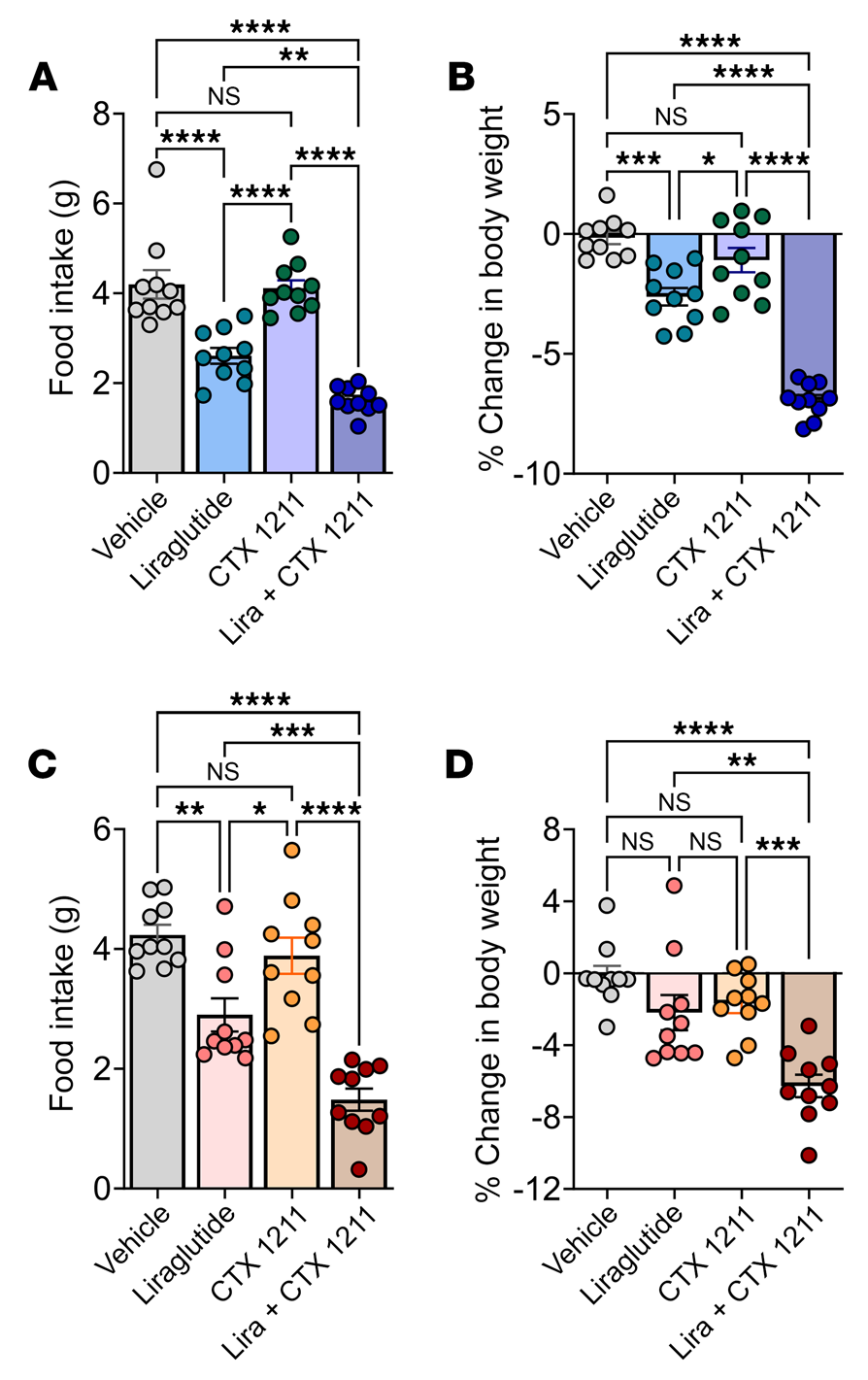
MC4R agonist synergistically increases the sensitivity of liraglutide. (**A** and **C**). Twenty-four-hour food intake and (**B** and **D**) body weight changes in response to vehicle (*n* = 10/group), liraglutide (0.1 mg/kg, *n* = 10/group), CTX 1211 (1 mg/kg, *n* = 10/group), and the combination of liraglutide plus CTX 1211 in male (**A** and **B**) and female(**C** and **D**) mice. Data represent the mean ± SEM. *P < 0.05, **P < 0.01, ***P < 0.001, and ****P < 0.0001, by 2-way ANOVA.

Earlier research showed that the MC3R presynaptically releases GABA from AgRP neurons that synapse onto downstream MC4R neurons, highlighting that the MC3R is a negative regulator of MC4R-expressing neurons (9). Furthermore, it has been reported that coadministration of the GLP1R analog liraglutide and the MC4R agonist setmelanotide has an additive effect on weight loss (29). Hence, we tested the hypothesis that the administration of MC4R agonists might also increase sensitivity to liraglutide-induced inhibition of food intake and weight loss (Figure 4, A–H). Peripheral administration of the MC4R peptide agonist CTX-1211 also increased the sensitivity to liraglutide-induced inhibition of food intake and weight loss in WT male mice 24 hours after treatment (Figure 4, A and B). Sensitization of feeding behavior was less evident in female mice (Figure 4C), but liraglutide-induced weight loss was clearly sensitized by CTX-1211 (Figure 4D). Setmelanotide (Imcivree) (32), an MC4R agonist marketed to treat certain forms of genetic obesity, resulted in similar responses with the combination of liraglutide (Figure 4, E–H), illustrating the idea that multiple MC4R agonists can increase the effect of liraglutide in inhibiting food intake and weight loss. Next, we tested whether MC3R deletion would further enhance sensitization of liraglutide action by CTX 1211 and setmelanotide. Vehicle, setmelanotide, or CTX 1211 (1.5 mg/kg, i.p.), liraglutide (0.1 mg/kg, i.p.), or both an MC4R agonist and liraglutide were administered to WT and *Mc3r^–/–^* mice, and 24-hour food intake and changes in body weight were measured (Figure 4, I–L). MC4R agonists and liraglutide decreased food intake and promoted weight loss in WT mice, the magnitude of which was increased for both agents in *Mc3r^–/–^* mice. Notably, the anorectic and weight loss responses to the coadministration of MC4R agonists and liraglutide were yet further increased in *Mc3r^–/–^* mice compared with WT mice (Figure 4, I–L). Next, we tested whether the heightened responsiveness observed with concurrent administration of the MC4R agonist and liraglutide was additive or synergistic. To test this, we treated WT mice with a low dose of CTX 1211 (1 mg/kg), a concentration known not to affect food intake or body weight, in combination with an optimal dose of liraglutide (0.1 mg/kg). Both male and female mice that received the cotreatment of liraglutide and CTX 1211 exhibited significant reductions in food intake and weight loss compared with those receiving liraglutide alone (Figure 5, A–D).

### Circuit mechanisms underlying anorectic hypersensitivity mediated by MC3R inhibition.

**Figure 6:**
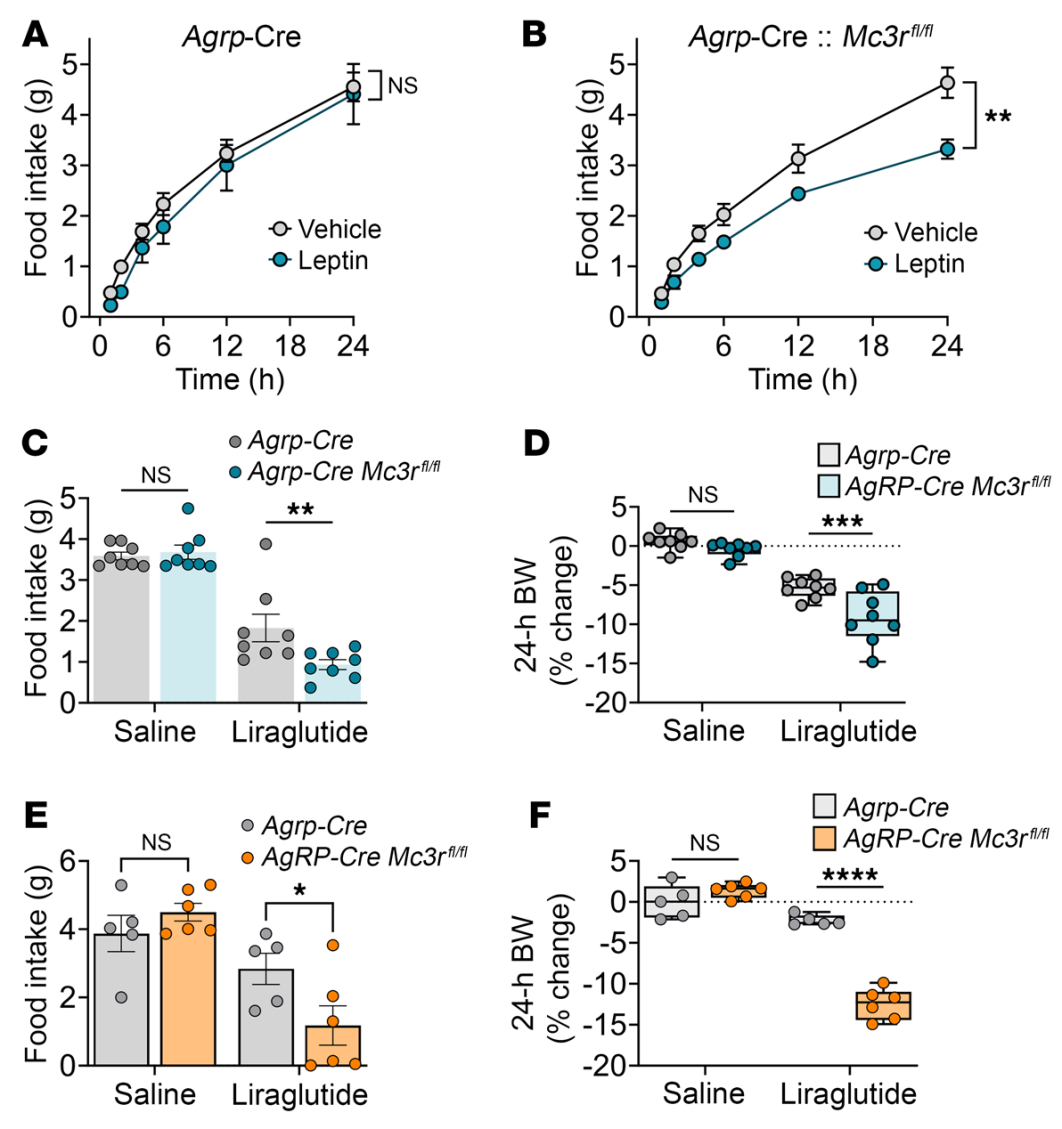
Specific deletion of *Mc3r* in AgRP neurons increases the responsiveness to liraglutide and leptin. (**A**) Time-course feeding for *Agrp-Cre* and (**B**) *Agrp-Cre* *Mc3r^fl/fl^* mice in response to leptin (1 mg/kg, i.p., *n* = 8/group). Twenty-four-hour food intake and change in body weight in (**C** and **D**) male and (**E** and **F**) female mice in response to liraglutide (0.1 mg/kg, s.c., *n* = 8/group/males, *n* = 4–6/group/females). Data represent the mean ± SEM. *P < 0.05, **P < 0.01, ***P < 0.001, and ****P < 0.0001, by 2-way, repeated-measures ANOVA with Tukey’s post hoc test (**A** and **B**) and 2-tailed Student’s *t* test (**C**–**F**).

**Figure 7:**
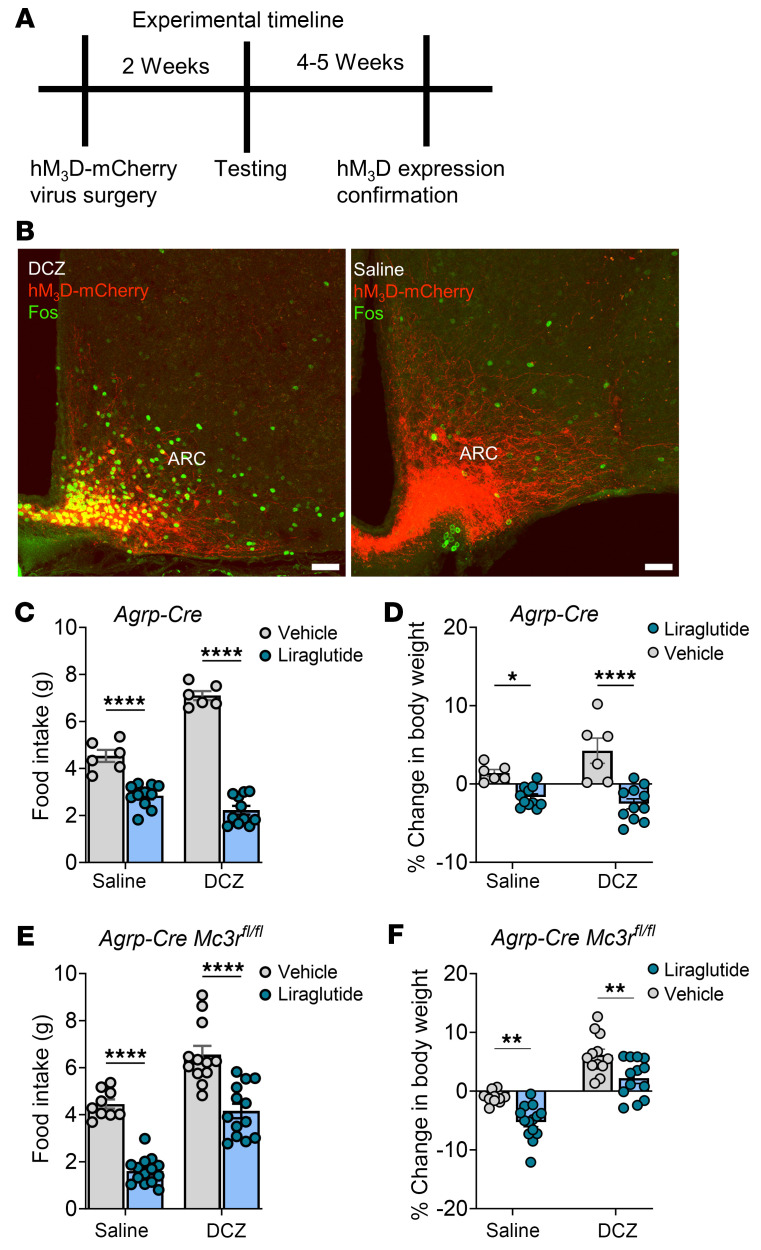
The reduction in food intake and weight loss induced by liraglutide persists with DCZ-mediated activation of hM3Dq-DREADD in AgRP neurons. (**A**) Experimental timeline for DREADD experiments. (**B**) AAV-mediated functional expression of hM3Dq-mCherry in AgRP neurons after DCZ (0.3 mg/kg) or saline treatment. Representative confocal images are shown after staining for c-Fos. Scale bars: 50 μm. (**C** and **E**) Twenty-four-hour food intake and (**D** and **F**) changes in body weight after liraglutide (0.1 mg/kg, s.c.) or vehicle treatment in the presence of DCZ (0.3 mg/kg, i.p.) or saline in *Agrp-Cre* and *Agrp-Cre* *Mc3r^fl/fl^* mice. Data are from both male and female mice and represent the mean ± SEM. *P < 0.05, **P < 0.01, and ****P < 0.0001, by 2-way, repeated-measures ANOVA with Tukey’s post hoc test (**A** and **B**) and 2-tailed Student’s *t* test (**C**–**F**).

**Figure 8:**
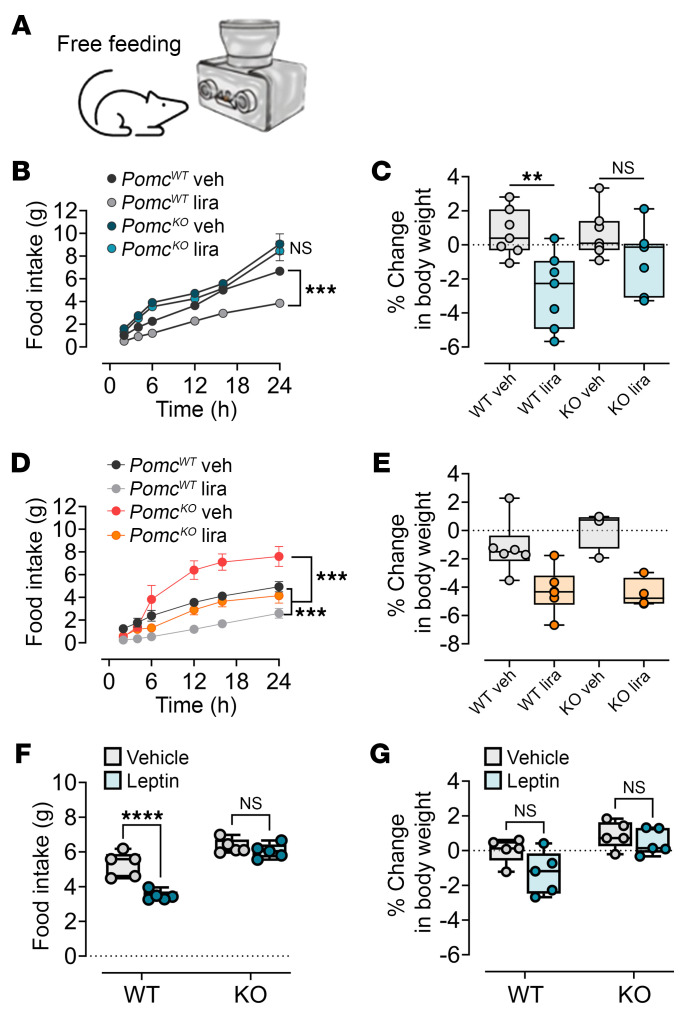
Genetic deletion of the neuronal *Pomc* gene abolishes the response to liraglutide and leptin in male mice. (**A**) Schematic of the FED3 feeding device in free-feeding mode. Twenty-four-hour food intake and changes in body weight 24 hours after injection of liraglutide in (**B** and **C**) male mice (0.2 mg/kg, s.c., *n* = 7/group) and (**D** and **E**) female mice (0.2 mg/kg, s.c., *n* = 4/group). (**F** and **G**) Twenty-four-hour food intake and body weight changes in response to leptin (3 mg/kg, i.p.) in male mice (*n* = 5/group). Data are expressed as the mean ± SEM. **P < 0.01, ***P < 0.001, and ****P < 0.0001, by repeated-measures 2-way ANOVA with Tukey’s post hoc analysis.

Previous studies have demonstrated that much of the physiological function of the MC3R derives from the expression of the receptor in AgRP neurons. The MC3R is expressed in nearly all AgRP neurons (10), which project widely throughout the CNS (11). For example, the defective fasting-induced activation of AgRP neurons essential for the stimulation of food intake, observed in the global *Mc3r^–/–^* mouse, is also seen in mice with deletion of *Mc3r* exclusively from AgRP neurons (13). Thus, the *Agrp-Cre* *Mc3r^fl/fl^* mouse was used to study the role of MC3R expression in AgRP neurons in the hypersensitivity to anorectic hormones described here. Male *Agrp-Cre* *Mc3r^fl/fl^* mice were more responsive to the anorectic effects of a single 1 mg/kg dose (i.p.) of leptin than were control *Agrp-Cre* mice (Figure 6, A and B). Male (Figure 6, C and D) and female (Figure 6, E and F) *Agrp-Cre* *Mc3r^fl/fl^* mice exhibited greater inhibition of feeding and greater weight loss, measured 24 hours following a 0.1 mg/kg (s.c.) dose of liraglutide than did control *Agrp-Cre* mice. These data suggest that hypersensitization to leptin and liraglutide can be recapitulated by MC3R loss from AgRP neurons alone. Given the impaired activation of AgRP neurons observed in both the global deletion of *Mc3r* and the selective deletion of *Mc3r* in AgRP neurons (13), we used a chemogenetics approach involving designer receptors exclusively activated by designer drugs (DREADDs) to assess the functional outcomes of specifically activating AgRP prior to liraglutide treatment. We hypothesized that in the presence of AgRP activation, we could partially rescue the hypersensitivity seen in response to liraglutide in mice with *Mc3r* deletion. We found that liraglutide-mediated food inhibition and weight loss persisted in mice following DREADD-mediated AgRP neuronal activation (Figure 7, A–D). However, mice with selective deletion of *Mc3r* in AgRP neurons and DREADD-mediated AgRP neuronal activation showed reduced responsiveness to liraglutide action on feeding and weight loss compared with similarly treated *Agrp-Cre* (control) mice (Figure 7, C–F). Finally, we examined the role of the *Pomc* gene, the source of the MC3R and MC4R agonist, in liraglutide and leptin action. Leptin and liraglutide directly activate pro-opiomelanocortin (POMC) neurons and also enhance presynaptic inputs to POMC neurons (12, 30). Leptin- and liraglutide-mediated inhibition of food intake and weight loss were significantly blunted in male mice with a neural-specific deletion of the *Pomc* gene; no effect was seen in female mice (Figure 8, A–G). While these data do not examine the role of POMC in the hypersensitization to liraglutide by MC3R ablation/antagonism, they do implicate an important role for melanocortin signaling in liraglutide action.

## Discussion

Previous studies have shown that the *Mc3r^–/–^* mouse exhibits modest, late-onset obesity (4), yet increased anorexia and weight loss in response to various challenges relative to WT littermates. For example, the *Mc3r^–/–^* mouse was demonstrated to exhibit greater anorexia and weight loss in response to LPS treatment or IL-1β treatment and greater loss of both fat mass and lean mass in response to implantation of Lewis lung carcinoma cells (6). More recently, we demonstrated that the *Mc3r^–/–^* mouse exhibit greater anorexia and weight loss than do WT mice in response to behavioral challenges, including restraint and social isolation (10). Furthermore, we demonstrated that the *Mc3r^–/–^* mouse exhibits a greater extent and duration of anorexia in response to a single dose of the MC4R agonist LY2112688 (9). Here, we sought to examine this phenomenon further and determine its potential utility in the pharmacotherapy of dietary obesity. First, careful dose-response curves demonstrated that the *Mc3r^–/–^* mouse had increased sensitivity to the anorectic and weight loss activities of the GLP1 agonists. In striking contrast, the *Mc3r^–/–^* mouse did not show increased sensitivity to the liraglutide-induced incretin effect or malaise, as indicated by a conditioned taste aversion assay. We have demonstrated that assays of Fos IHC, used as an indicator of neuronal activation, identified a liraglutide-induced increase in Fos signal in multiple hypothalamic feeding centers, but no increase in a region mediating emesis, the AP, in *Mc3r^–/–^* versus WT mice. Since mice do not show measurable emesis, it will be important to assess whether MC3R antagonism can blunt the emesis induced by the GLP1 drugs in a susceptible species or if comparable weight loss can be obtained at lower GLP1 drug concentrations by MC3R antagonist treatment to avoid pharmacotherapy-induced side effects.

Importantly, the hypersensitization to GLP1 drugs could also be demonstrated using the MC3R antagonist C11 in acute (24-hour) and chronic (3-day) treatment models. Thus, we expect that most of the anorectic hypersensitization phenomena do not result from developmental consequences of MC3R deletion and may ultimately be replicated pharmacologically. Unfortunately, the C11 molecule is difficult to synthesize and has limited brain penetrance, thus requiring i.c.v. administration. To our knowledge, a potent, receptor subtype–specific MC3R antagonist with brain penetrance has yet to be described.

To determine whether the hypersensitivity phenomenon was restricted to GLP1 analogs, we next tested the responsiveness of the *Mc3r^–/–^* mouse to both the slow-acting adipostatic hormone leptin and the rapid, acute-acting satiety factors PYY_3–36_ and CCK. The *Mc3r^–/–^* mouse is obese and hyperleptinemic and thus should exhibit leptin resistance. Remarkably, the *Mc3r^–/–^* mouse showed increased sensitivity to leptin relative to WT littermates, as determined by 24-hour food intake and weight-loss monitoring. Moreover, pharmacological inhibition of the MC3R also increased leptin action by decreasing food intake and promoting weight loss in males and females (data not shown). Additional work will be needed to understand how MC3R antagonism disrupts the well-characterized phenomenon of leptin resistance. Interestingly, the *Mc3r^–/–^* mouse was also hypersensitive to the acute inhibition of feeding by the satiety factors PYY_3–36_ and CCK.

We have demonstrated that MC3R acts postsynaptically on AgRP neurons to negatively regulate MC4R neurons in PVH (9) and POMC neurons in the arcuate nucleus (12). However, these same AgRP and POMC neurons project throughout the CNS to a wide variety of centers known to regulate feeding (31–36) and to behavioral sites known to be suppressed in response to hunger and weight loss (37–39). Thus, the data here support the hypothesis that the MC3R provides negative feedback affecting circuits controlling a wide variety of hormonal and behavioral inputs to feeding and energy homeostasis. The studies shown here suggest that much of the hypersensitization phenomenon may be mediated by the disruption of MC3R signaling in AgRP neurons alone. Moreover, activating AgRP neurons through a chemogenetic approach demonstrated a partial blunting of the reduced food intake induced by anorectic drugs in mice lacking the MC3R in AgRP neurons. Our studies show that MC3R antagonism may also enhance the weight-loss activity of the MC4R agonist setmelanotide. Whether this is due to the added effects of GABA and NPY released onto MC4R neurons in addition to MC4R agonism by setmelanotide or to action on secondary sites of MC3R expression outside of AgRP neurons remains to be determined. The primary melanocortinergic AgRP, POMC neurons, and MC4R neurons are likely to play a role in the anorexigenic hypersensitivity resulting from loss of MC3R signaling, and the hypersensitivity to GLP1 agonist liraglutide can be reproduced with the MC4R agonists setmelanotide and CTX-1211; indeed, subthreshold doses of CTX-1211, having no effect on food intake or weight loss, reproduced the phenomenon. Neural deletion of *Pomc* significantly blunted liraglutide-induced anorexia and weight loss in male mice. However, we did not examine the hypersensitization phenomenon in this model.

Rupp et al. demonstrated that neurons in the DMH are sensitive to both leptin and liraglutide (40). They further found the reactivation of *glp1r*, specifically in neurons expressing the leptin receptor, was adequate for liraglutide to effectively suppress food intake. Our observation of pronounced Fos activation in the medial basal hypothalamus (MBH) of *Mc3r^–/–^* mice after liraglutide treatment suggests the presence of MC3R-regulated neurons in the MBH that may be responsive to anorexigenic agents such as liraglutide and leptin. Furthermore, significant sexual dimorphism of expression of the MC3R has been reported (8, 10, 11), and some sexually dimorphic responses to anorexigenic agents were observed in the present study as well. Additional work will need to be completed to determine the specific circuit mechanisms underlying the role of the MC3R in sensitization to both behavioral challenges reported previously and the wide variety of anorexigenic hormones and drugs reported here.

In conclusion, our results indicate that the loss or pharmacological inhibition of MC3R can reliably hypersensitize animals to GLP1R agonists without promoting malaise or peripheral incretin effects. Furthermore, MC3R loss promotes generalized hypersensitivity to multiple anorexigenic agents, including CCK and leptin, which are short- and long-term satiety peptides, respectively. Removal of the tonic inhibition of anorexic circuits mediated by the MC3R may be similarly achieved by subthreshold stimulation of MC4R neurons, although more effective MC3R antagonists will be needed to test these 2 approaches in parallel. Thus, melanocortin compounds may have utility in improving the pharmacotherapies available to treat obesity and other metabolic disorders.

## Methods

### Sex as a biological variable.

Previous studies of the MC3R produced sex-dimorphic processes, including physiology and anatomy (10, 11). As such, male and female mice were used in the present study. The sex-dimorphic findings in this study are noted; however, the underlying differences between males and females in their responses to anorectic stimuli require further studies. Statistical analyses were performed but did not take sex into account, and each respective sex was analyzed separately. Additionally, this study lacks sufficient statistical power to analyze sex as a stratified variable if data were analyzed together.

### Mice.

Experiments were performed on C57BL/6J (WT) mice purchased from The Jackson Laboratory (JAX), transgenic *Mc3r* WT (*Mc3r^+/+^*) and KO (*Mc3r^–/–^*) mice (bred in-house), and neural-specific *Pomc*-KO (*nPomc*KO) (41). The n*Pomc*KO mice were provided by Malcom Low (University of Michigan School of Medicine, Ann Arbor, Michigan, USA). *Agrp-Cre* were purchased from JAX (strain no. 012899) and crossed with *Mc3r^fl/fl^* mice (13) (bred and generated in-house). *Agrp-Cre* *Mc3r^fl/fl^* mice were previously validated (13). Mice were maintained on a reverse 12-hour light/12-hour dark schedule and given ad libitum access to chow and water unless otherwise specified. Both sexes were used in these experiments and are shown as such in the results.

### Peptides and drugs.

Liraglutide (0.05–0.4 mg/kg, s.c., Tocris), semaglutide (1–100 nmol/kg, s.c., Cayman Chemical), CCK octapeptide (sulfated) (2 and 4 μg/kg, i.p., Tocris), and PYY3-36 were dissolved in a USP-grade sterile PBS, and working concentrations were made from stock on the day of the experiment. Leptin (0.1– 1 mg/kg, i.p., Golden West BioSolutions) was dissolved in sterile isotonic saline, and working concentrations were made on the day of the experiment. Tirzepatide hydrochloride (1–5 nmol/kg, s.c., MedChem Express) was dissolved in DMSO to create stock solutions, and working concentrations were made in PBS. Setmelanotide (1.5 mg/kg, i.p., Rhythm Pharmaceuticals) was dissolved in saline, and working concentrations were made on the day of the experiment. Compound 11 (15) (0.5–1 nmol, i.c.v.) was dissolved in DMSO. Compound CTX-1211 [Ac-Arg-*cyclo*(Cys-d-Ala-Arg-d-Phe-Arg-Trp-Cys-NH_2_); 2 mg/kg, i.p., unless otherwise indicated] is an MC4R agonist peptide (EC_50_ = 3.8 × 10^–10^ M) that was provided by Courage Therapeutics Inc. and dissolved in saline. Fresh concentrations were made on the day of the experiment.

### Acute and chronic nighttime feeding.

All mice (9–14 weeks old) were single-housed in individual cages, acclimated to injection, and handled daily for 5–7 days before experimentation. Mice were randomly assigned to groups on the first day of the experiment. The number of mice used in each experiment is given in the figure legends. The same mice were used in a crossover manner for the dose-response experiments. There were at least 4 days between each dosage. All compounds were administered 20–30 minutes before the onset of the dark, active cycle. Food was manually weighted according to the experimental time points. For chronic experiments, mice received a vehicle injection for at least 4 days before the start of the experiment. Compounds/peptides were injected in a volume of 150–200 μL/injection. For the CCK and PYY_3–36_ experiments, preweighed chow pellets were measured before and after the acute feeding test. The n*Pomc*-KO mice exhibit hyperphagia and produce excess food crumbs, making it difficult to measure food intake accurately. To address this, we used the Feeding Experimentation Device (FED3) system (42) with pelleted standard chow to measure food intake for these mice. We did not observe any significant increase in chewing or crumbs in the bedding with the FED3 system.

### Surgery and cannula placement.

C57BL/6J mice (7–8 weeks old) were housed in groups of 3 with ad libitum access to standard chow and water before surgery. On the day of surgery, mice were anesthetized with 3%–4% (v/v) isoflurane before being placed in a stereotaxic surgical frame (Kopf) and then maintained at 1.5%–2% (v/v) isoflurane for the rest of the surgery. Cannulae were implanted as previously described (10). Guide Cannulae (Plastics One) targeted the right lateral ventricle using the following flat skull coordinates (–0.460 mm posterior to bregma, 1.00 lateral to the midline, and –2.20 ventral to the surface of the skull). The cannula was secured to the skull with dental cement. Following recovery, mice received an i.c.v. injection of 20 ng angiotensin II (MilliporeSigma) to confirm cannula placement by angiotensin-induced water intake. Cannula placement was also confirmed postmortem under a microscope.

### Stereotaxic microinjection of adeno-associated virus into the ARH.

Intracranial stereotaxic surgery was performed as described above in *Agrp-Cre* and *Agrp-Cre Mc3r^fl/fl^* mice (8–10 weeks old). Chemogenetic virus delivery was performed as described previously (10). Briefly, adeno-associated virus (AAV) viral vectors [AAV5 hSyn-DIO-hM3D(Gq)-mCherry, Addgene] were bilaterally injected (200 nL) into the ARH using a micromanipulator (Narishige) attached to a pulled glass pipette. The ARH coordinates used were −1.46 mm posterior to bregma, ±0.3 mm lateral to the midline, and –5.80 ventral to the surface of the skull. To ensure full penetration of the AAV into the ARH, the glass pipette was left in place for 8–10 minutes before being removed, and the skin was sutured. Mice were then kept on a warming pad until alert and mobile. The DREADDs actuator deschloroclozapine (DCZ) (0.3 mg/kg, Tocris) or vehicle (1% DMSO in 99% saline) was used to activate hM3Dq-expressing AgRP neurons. At the completion of the study, mice were anesthetized in an isoflurane drop jar and transcardially perfused with 1× PBS followed by 4% paraformaldehyde. Brains were then removed and postfixed in 4% paraformaldehyde and sectioned into 50 μm slices containing the ARH. Injection sites were confirmed in all mice by evaluating the expression of mCherry in the ARH under a fluorescence microscope. Only mice with mCherry expression were included in the final analysis.

### Saccharin-conditioned taste aversion.

Age-matched WT and *Mc3r^–/–^* mice were handled and individually housed for 1 week with ad libitum access to standard chow and water. A 2-bottle test was used to administer the saccharin solution and water to mice. For the bottles, custom-made bottles (25 mL serological pipette) were fitted with rubber stoppers on one end and fabricated sipper tubes with a hole diameter of 3.175 mm (~0.12 inches) on the other end. Silicone tubing was used to seal the sipper tube and the bottle. Mice were acclimated to the 2-bottle choice and the timing of the presentation. The bottles were placed in the home cage of the mice for 2 days, 4–6 hours after the dark cycle, and mice received an i.p. injection of sterile saline during this 2-day period. Bottles were measured to ensure the mice were drinking equally from the 2 bottles. Following acclimation, the mice were conditioned for 2 days, and access to water was removed. On the conditioning days, 1 group of mice received 1 mL intra-oral application of 0.1% saccharin made in water (conditioned stimulus [CS]) and then immediately paired with i.p. injections of either 150 mM LiCl to cause gastric malaise (unconditioned stimulus), 0.05 mg/kg liraglutide, or 150 mM NaCl (saline) as a control solution. Mice were then given access to the saccharin solution and water in the 2-bottle tests for an additional 90 minutes. On the 2 testing days, the mice were given access to the 2-bottle test during the typical 90-minute access; 1 bottle had water, and the other bottle had a saccharin solution. The bottles (left vs. right) were switched at the 45-minute mark to account for potential sidedness. An empty cage with a 2-bottle test was set up to account for possible spillage and was thus subtracted from each intake. Fluid intake was recorded at the end of the 90-minute test.

### Immunofluorescence staining.

Mice were handled and injected s.c. with 150 μL sterile saline daily for 4 days before the experiment to minimize background neuronal activation (Fos) due to stress. Immunofluorescence experiments were conducted to mimic the first 90 minutes of the acute experiments; thus, mice were injected with the compounds at the start of the dark cycle to simulate the drugs’ anorectic effects. Mice received a s.c. injection of 0.1 mg/kg liraglutide or vehicle (PBS). After 90 minutes, mice were given a lethal dose of anesthesia and transcardially perfused with 4% paraformaldehyde in 0.1 M phosphate buffer (pH 7.4). Brains were removed and postfixed in the same fixative overnight. Tissues were rinsed in PBS and cryoprotected in 30% sucrose in PBS until the tissue sank. Brains were embedded in OCT (Tissue-Tek), frozen in a dry ice ethanol bath, and stored at –80°C. Hypothalamic and brainstem sections were cut on a cryostat at 50 μm thickness. The tissues were immediately washed with PBS and rocked slowly at room temperature (RT) to warm. Free-floating sections were blocked for 1 hour with 0.3% Triton and 5% normal serum in PBS. Primary cFos antibody (1:2,000; Synaptic Systems, 226 004) was diluted in the blocking buffer. Sections were incubated with a primary antibody overnight, rocked at RT, rinsed 3 times in PBS, and incubated with a secondary antibody in blocking buffer (Alexa Fluor–conjugated antibodies, Thermo Fisher Scientific, 1:500) for 2 hours at RT. For the control experiments, the primary antibody was omitted, and sections were incubated with the blocking buffer instead. Sections were rinsed 3 times with PBS, counterstained with Hoechst 33342 (Thermo Fisher Scientific, 1:2,000 in PBS-0.05% Tween 20), and rinsed 2 times with PBS. Sections were moved, and slides and coverslips were mounted with Fluoromount G (Southern Biotechnology).

### Glucose tolerance tests.

Mice were fasted at the onset of the light cycle for 6 hours and randomly received either treatment (0.2 mg/kg liraglutide) or vehicle (PBS). Repeated oral GTTs were given 2 weeks apart to ensure proper washout and recovery. Baseline blood was collected from the tail, followed by oral administration of 0.5–2 g/kg glucose bolus in water. Blood glucose was assayed over a 2-hour period. All mice received saline injections to account for fluid loss at the end of the experiment, and food was returned to the mice.

### Statistics.

The present study did not use a specific tool to predetermine sample sizes but relied on previous experiences to determine the number of biological replicates. The sample sizes are present in the figure legends, and a minimum of 5 animals were used for each treatment to reach statistical significance. Statistical analyses were performed and graphs designed and generated using GraphPad Prism (GraphPad Software). The experimental values in the figures represent the mean ± SEM. Statistical significance between 2 groups or more were determined using a 2-tailed Student’s *t* test and 2-way ANOVA with Tukey’s multiple-comparison test. A *P* value below 0.05 was deemed to indicate statistical significance.

### Study approval.

The IACUC of the University of Michigan approved all animal procedures following the American Veterinary Medical Association guidelines.

### Data availability.

All supporting data from the study can be found in the Supporting Data Values file.

## Author contributions

NSD, PRS, and RDC designed the research studies. NSD, YG, YW, and AAJR conducted the experiments and acquired the data. NSD and RDC analyzed the data. SYW, LEG, TKS, STJ, and AKM provided reagents. NSD and RDC wrote the manuscript.

## Supplementary Material

Supplemental data

Supporting data values

## Version 1 07/15/2024

Electronic publication

## References

[1] Huszar D (1997). Targeted disruption of the melanocortin-4 receptor results in obesity in mice. Cell.

[2] Lam BYH (2021). MC3R links nutritional state to childhood growth and the timing of puberty. Nature.

[3] Vaisse C (1998). A frameshift mutation in human MC4R is associated with a dominant form of obesity. Nat Genet.

[4] Butler A (2000). A unique metabolic syndrome causes obesity in the melanocortin-3 receptor-deficient mouse. Endocrinology.

[5] Chen AS (2000). Inactivation of the mouse melanocortin-3 receptor results in increased fat mass and reduced lean body mass. Nat Genet.

[6] Marks D, Cone RD (2003). The role of the melanocortin-3 receptor in cachexia. Ann N Y Acad Sci.

[7] Renquist BJ (2012). Melanocortin-3 receptor regulates the normal fasting response. Proc Natl Acad Sci U S A.

[8] Lippert RN (2014). Gender-specific roles for the melanocortin-3 receptor in the regulation of the mesolimbic dopamine system in mice. Endocrinology.

[9] Ghamari-Langroudi M (2018). Regulation of energy rheostasis by the melanocortin-3 receptor. Sci Adv.

[10] Sweeney P (2021). The melanocortin-3 receptor is a pharmacological target for the regulation of anorexia. Sci Transl Med.

[11] Bedenbaugh MN (2022). Organization of neural systems expressing melanocortin-3 receptors in the mouse brain: Evidence for sexual dimorphism. J Comp Neurol.

[12] Cowley MA (2001). Leptin activates anorexigenic POMC neurons through a neural network in the arcuate nucleus. Nature.

[13] Gui Y (2023). Melanocortin-3 receptor expression in AgRP neurons is required for normal activation of the neurons in response to energy deficiency. Cell Rep.

[14] Rubino D (2021). Effect of continued weekly subcutaneous semaglutide vs placebo on weight loss maintenancein adults with overweight or obesity: The STEP 4 Randomized Clinical Trial. JAMA.

[15] Singh A (2013). Structure-activity relationships of peptides incorporating a bioactive reverse-turn heterocycle at the melanocortin receptors: identification of a 5800-fold mouse melanocortin-3 receptor (mMC3R) selective antagonist/partial agonist versus the mouse melanocortin-4 receptor (mMC4R). J Med Chem.

[16] Coskun T (2018). LY3298176, a novel dual GIP and GLP-1 receptor agonist for the treatment of type 2 diabetes mellitus: From discovery to clinical proof of concept. Mol Metab.

[17] Jastreboff AM (2022). Tirzepatide once weekly for the treatment of obesity. N Engl J Med.

[18] El K (2023). The incretin co-agonist tirzepatide requires GIPR for hormone secretion from human islets. Nat Metab.

[19] Muller TD (2019). Glucagon-like peptide 1 (GLP-1). Mol Metab.

[20] Anderson J (2018). The pharmacokinetic properties of glucagon-like peptide-1 receptor agonists and their mode and mechanism of action in patients with type 2 diabetes. J Fam Prac.

[21] Astrup A (2012). Safety, tolerability and sustained weight loss over 2 years with the once-daily human GLP-1 analog, liraglutide. Int J Obes (Lond).

[22] Buse JB (2009). Liraglutide once a day versus exenatide twice a day for type 2 diabetes: a 26-week randomised, parallel-group, multinational, open-label trial (LEAD-6). Lancet.

[23] Kanoski SE (2012). The role of nausea in food intake and body weight suppression by peripheral GLP-1 receptor agonists, exendin-4 and liraglutide. Neuropharmacology.

[24] Borner T (2022). Glucagon-like peptide-1 in diabetes care: Can glycaemic control be achieved without nausea and vomiting?. Br J Pharmacol.

[25] Young C (2020). Diabetic gastroparesis: a review. Diabetes Spectr.

[26] Halatchev IG, Cone RD (2005). Peripheral administration of PYY(3-36) produces conditioned taste aversion in mice. Cell Metab.

[27] Yamamoto H (2003). Glucagon-like peptide-1-responsive catecholamine neurons in the area postrema link peripheral glucagon-like peptide-1 with central autonomic control sites. J Neurosci.

[28] Myers MG (2010). Obesity and leptin resistance: distinguishing cause from effect. Trends Endocrinol Metab.

[29] Clemmensen C (2015). Dual melanocortin-4 receptor and GLP-1 receptor agonism amplifies metabolic benefits in diet-induced obese mice. EMBO Mol Med.

[30] He Z (2019). Direct and indirect effects of liraglutide on hypothalamic POMC and NPY/AgRP neurons - implications for energy balance and glucose control. Mol Metab.

[31] Wang D (2015). Whole-brain mapping of the direct inputs and axonal projections of POMC and AgRP neurons. Front Neuroanat.

[32] Jais A, Bruning JC (2022). Arcuate nucleus-dependent regulation of metabolism-pathways to obesity and diabetes mellitus. Endocr Rev.

[33] Cone RD (2005). Anatomy and regulation of the central melanocortin system. Nat Neurosci.

[34] Rau AR, Hentges ST (2019). GABAergic inputs to POMC neurons originating from the dorsomedial hypothalamus are regulated by energy state. J Neurosci.

[35] Garfield AS (2016). Dynamic GABAergic afferent modulation of AgRP neurons. Nat Neurosci.

[36] Berrios J (2021). Food cue regulation of AGRP hunger neurons guides learning. Nature.

[37] Palmiter RD (2018). The parabrachial nucleus: CGRP neurons function as a general alarm. Trends Neurosci.

[38] Chen Y (2016). Hunger neurons drive feeding through a sustained, positive reinforcement signal. Elife.

[39] Li HE (2021). Hypothalamic-extended amygdala circuit regulates temporal discounting. J Neurosci.

[40] Rupp AC (2023). Suppression of food intake by Glp1r/Lepr-coexpressing neurons prevents obesity in mouse models. J Clin Invest.

[41] Bumaschny VF (2012). Obesity-programmed mice are rescued by early genetic intervention. J Clin Invest.

[42] Matikainen-Ankney BA (2021). An open-source device for measuring food intake and operant behavior in rodent home-cages. Elife.

